# Spatio-temporal evolution laws of storage coefficient of coal mine underground reservoir and contact network of crushed rock

**DOI:** 10.1371/journal.pone.0293611

**Published:** 2023-10-30

**Authors:** Xuan Qin, Zhiguo Cao, Lichang Wei, Huan Yang, Hao Sun, Shenggui Zhou

**Affiliations:** 1 State Key Laboratory of Water Resource Protection and Utilization in Coal Mining, Beijing, China; 2 China Academy of Safety Science and Technology, Beijing, China; 3 School of Civil and Resource Engineering, University of Science and Technology Beijing, Beijing, China; Xi’an University of Science and Technology, CHINA

## Abstract

Spatio-temporal evolution laws of storage coefficient and contact network of crushed rock are of great significance for the construction and utilization of underground reservoirs in coal mines. Based on discrete element method and irregular rigid block model, spatio-temporal evolution laws of storage coefficient and contact network of crushed rock under different overburden stresses are investigated and the following main conclusions are obtained: (1) The average storage coefficient and the storage coefficient at different vertical heights of the crushed rock packing system decrease exponentially as the overburden stress increases. When the overburden stress ranges from 0 to 20 MPa, the average storage coefficient decreases by 48.947%. (2) The average void radius and throat radius of water storage space decrease exponentially as the overburden stress increases. The increase in overburden stress leads to the transformation of large voids into smaller voids, causing a gradual decrease in void connectivity and a tendency towards irregular void shapes. (3) With the increase of overburden stress, the number of strong contacts in the packing system of crushed rock increases and gradually expands from the top to the bottom. The average contact force of crushed rock increases exponentially, while the coordination number increases linearly. (4) As the overburden stress increases, the majority of contact directions are concentrated within the ±30° range in the loading direction. This increase results in an enhancement of the anisotropy of the packing system structure of crushed rock.

## 1 Introduction

Utilizing the goaf formed by coal mining to establish an underground coal mine reservoir is a crucial approach for addressing the challenge of achieving coordinated development between large-scale, high-intensity coal mining and water resources protection in western China [1]. The space between crushed rocks in the goaf serves as the primary water storage area in underground reservoirs in coal mines. As the coal mining face advances, the overburden load on the crushed rocks in the goaf gradually increases, leading to continuous compaction and reduction of voids. This process results in significant variations in the spatial and temporal distribution and evolution of the storage coefficient in the goaf [2–6]. In recent years, there have been many qualitative studies but few quantitative studies on the storage coefficient of underground reservoirs. However, this trend has overlooked the crucial role played by the structure and mechanical properties of the packing system of crushed rock in understanding the water storage space structure. Consequently, our understanding of the water storage performance of underground reservoirs in coal mines remains limited, particularly hindering the application of underground water storage technology in coal industries in western China. Therefore, it is of utmost significance to investigate the spatio-temporal evolution laws of the storage coefficient of coal mine underground reservoirs and the contact network of crushed rock. This research will greatly contribute to the construction and utilization of coal mine underground reservoirs.

Numerous scholars have conducted extensive research on the storage coefficient of underground reservoirs in coal mines. This research has been conducted through various methods such as theoretical analysis, laboratory experiments, and numerical simulations. In theoretical analysis research, Liang et al. [7] constructed a non-uniform continuous distribution model of storage coefficient and permeability of crushed rock based on the subsidence theory of roof strata. Wang et al. [8] constructed a three-dimensional void distribution model through theoretical derivation, and found that the storage coefficient is in a "shovel" shape, with a large storage coefficient in the shallow part and on both sides of the roadway, and a small storage coefficient in the middle and inside. Fang et al. [9] established a calculation model for water storage coefficient considering the effect of effective stress and determined the analytical solution of the model. In laboratory experiment research, Pappas et al. [10] found that the stress-strain curve of crushed rocks during compaction was nonlinear. Ma et al. [11] and Su et al. [12] studied the variation trends of void distribution of broken rocks such as sandstone, mudstone and sandy mudstone in goaf under compression by using a special device together with the MTS815.02/ RMT-150B Rock Mechanics Test System. Chen et al. [13] studied the influence of water saturation on the compaction characteristics of crushed rock in the coal seam roof, and the research results showed that the rock strength is negatively correlated with the reduction of residual swelling coefficient, residual void ratio and compaction degree. Zhang et al. [14] and Feng et al. [15] investigated the relationship between void ratio and stress during the compaction process of large crushed rock and continuous graded saturated crushed rock, using a self-made rock bearing deformation device. In numerical simulation research, Zhang et al. [16] used the discrete element software PFC and spherical particle model to study the stress, void structure and fracture evolution characteristics of crushed coal samples in caving zone during compaction. Hu et al. [17] simulated the compaction process of crushed rocks in goaf based on the discrete element method, and found that the external load could not be transferred evenly from the top to the bottom of crushed rocks. Hu et al. [18] found that the stress in the packing system of crushed rock is mainly transmitted downward through the strong force chain, and the void ratio in the lower part of packing system of crushed rock was the highest. Zhang et al. [19] quantitatively analyzed the evolution characteristics of stress and void ratio of crushed rock during compression, and described the stress evolution and fracture evolution characteristics of crushed rock at each loading stage.

The current research on storage coefficient and water storage space of underground reservoirs in coal mines primarily relies on empirical methods, indoor or mine pumping and draining tests, or theoretical calculations. However, these research methods have limitations in studying the meso-structure and mechanical characteristics of water storage space due to technical constraints. As a result, the existing research results do not provide effective guidance for the design and planning of the actual underground reservoir capacity. By utilizing a parametric model of a solid particle system, the discrete element method enables simulation and analysis of the mechanical behavior of particles. This approach offers significant advantages in investigating particle interactions and other related issues. It accurately simulates the process of crushed rock packing and allows for the analysis of the formation and evolution of the spatial structure of water storage space in goaf. In this study, a numerical packing model of crushed rock is established using the discrete element software PFC3D and the Rigid Block Model (RBM). The model is used to examine the spatio-temporal evolution laws of the storage coefficient of a coal mine underground reservoir under different overburden stresses, and to reveal the evolution mechanism from the perspective of the contact network of crushed rock. The primary objective and innovation of this study is to conduct basic research on the relationship between the meso-structure characteristics of underground reservoir water storage space and the meso-contact network of irregular crushed rock under different overburden stresses by using the rigid block model, which can reliably characterize the shape characteristics of irregular particles while ensuring high simulation efficiency. However, it is important to note that this study does not aim to comprehensively explore all the factors that affect the storage coefficient or determine the detailed design parameters of underground reservoirs in coal mines. Factors such as particle gradation, strength, water absorption characteristics, and coal seam occurrence conditions have not been specifically considered in this research.

## 2 Test schemes

After obtaining the grading and shape distribution characteristics of real crushed rocks, this study uses the three-dimensional RBM in PFC3D to conduct numerical compression tests on crushed rock. The study also investigates the spatio-temporal evolution laws of the storage coefficient of coal mine underground reservoir and the contact network of crushed rock under different overburden stresses. The design and procedures of these numerical compression tests are described as follows:

### 2.1 Grading and shape distribution characteristics of crushed rocks

**Fig 1:**
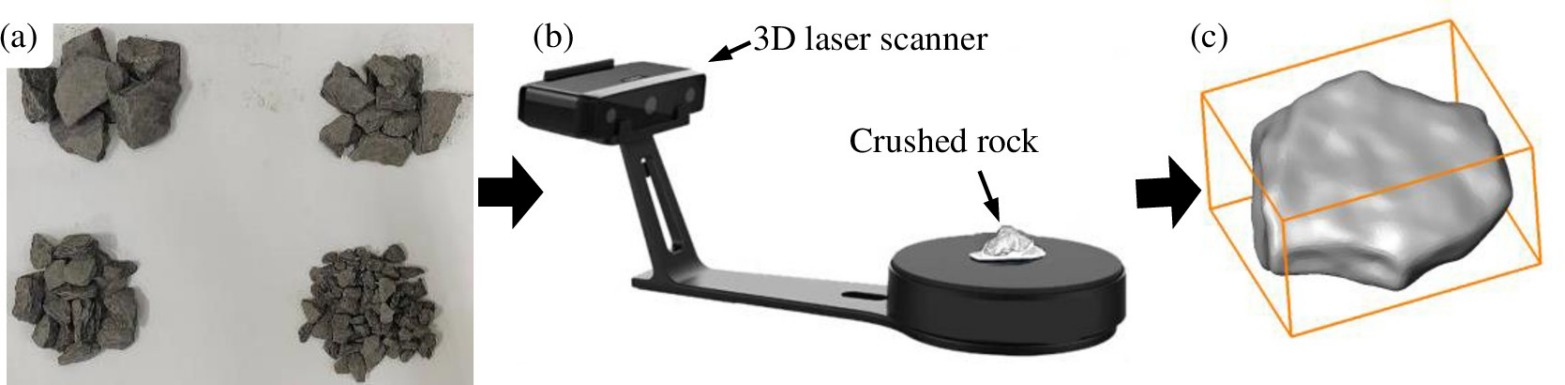
3D model construction processes of crushed rock: **a** real crushed rock, **b** 3D laser scanning of crushed rock, **c** 3D model of crushed rock.

Grain grading and shape distribution characteristics are crucial factors that impact the structure and mechanical properties of particle packing systems [20, 21]. As a result, it is essential to quantitatively assess the grading and shape of crushed rocks in the initial stages. Crushed rocks used here are taken from Halagou Coal Mine, Daliuta Town, Shenmu County, Shaanxi Province, with a particle size range of 5 to 45 mm and a density of 2300 kg/m^3^. In this paper, 1200 crushed rocks are randomly selected and scanned with an EiScan-SE 3D laser scanner equipped with the SHINING 3D software to obtain 3D model of crushed rock. The 3D model construction process of crushed rock mass is shown in Fig 1.

In order to accurately describe the size of crushed rock, it is inappropriate to directly use the sieving particle size due to its irregular shape. Therefore, this study introduces the concept of equivalent particle size *D*, which represents the spherical particle diameter that has the same volume as the measured particle. Previous methods for characterizing the shape of real particles have focused on overall shape, angular features, and surface texture, using multiple shape indicators [22]. However, these methods do not facilitate establishing a quantitative relationship between particle shape and its physical and mechanical properties [23]. To address this, this paper proposes the use of a dimensionless indicator *M* [24] to characterize the three-dimensional particle shape. This indicator takes into account both the first and second scale particle shape parameters, providing a comprehensive characterization of particle shape. Its reliability has also been verified in previous studies [23]. The equation of shape coefficient *M* is shown in Eq (1):

M=(A/V×D)/6 (1)

Here, *A* is the particle surface area and *V* is the particle volume. The *M* value of spherical particle is 1, while the *M* value of non-spherical particle is greater than 1. The more irregular the particle is, the greater the *M* value is.

**Fig 2:**
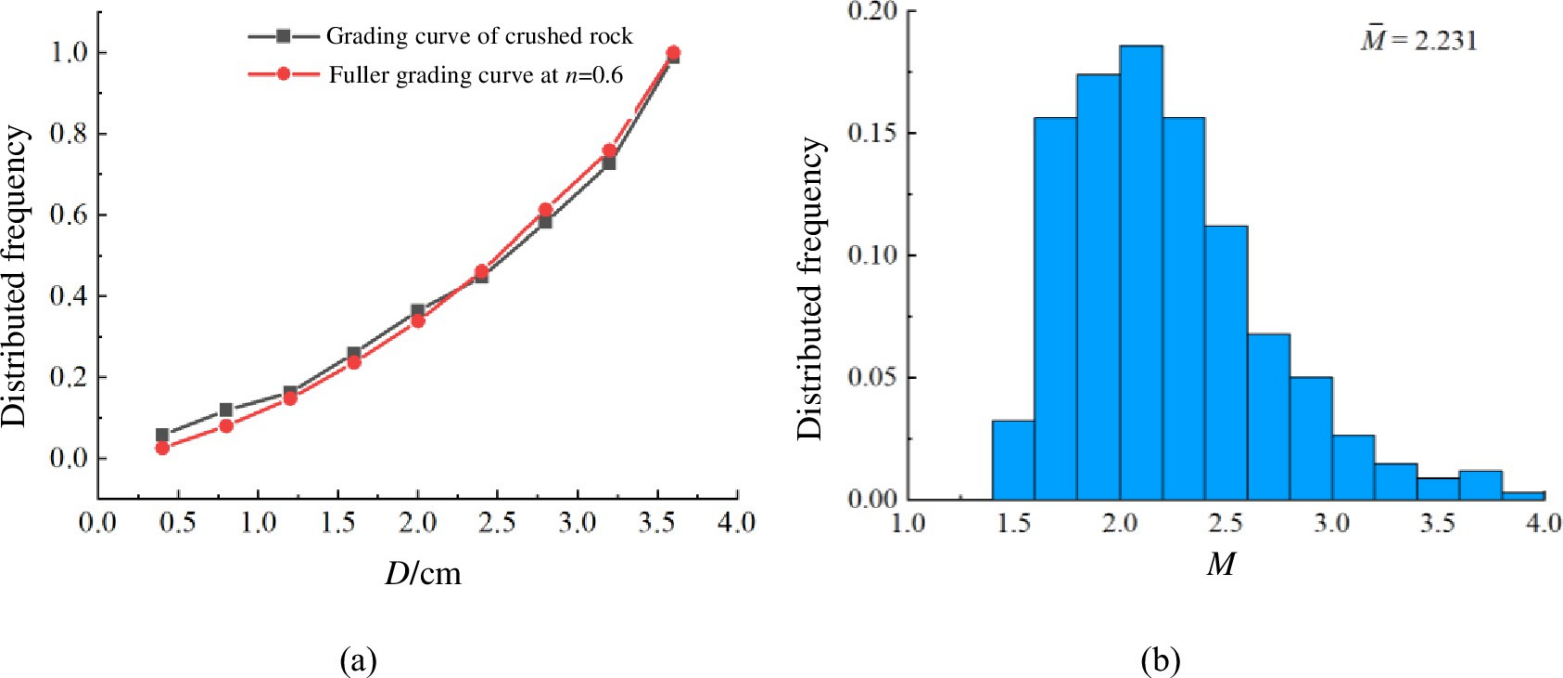
Statistical distributions of equivalent particle size *D* and shape coefficient *M* of crushed rock: **a** equivalent particle size *D*, **b** shape coefficient *M*.

By employing scanning and constructing techniques, the MATLAB software enables the quantitative characterization of crushed rock’s equivalent particle size *D* and shape coefficient *M*. The results are shown in Fig 2. The curve *n* = 0.6 in Fig 2(A) is the result determined by Fuller grading equation (Eq (2)) [25].

$$P=100\times {\left(\frac{d}{d_{\max}}\right)}^{n}$$ (2)

Here, *P* is the passing percentage of rigid block on particle size *d*, *d*_max_ is the maximum particle size, and *n* is the Talbol power index. The analysis of Fig 2(A) and 2(B) reveals that the grading distribution of crushed rocks aligns closely with the Fuller grading curve when *n* = 0.6. This indicates that the grading of rigid blocks in the numerical tests is determined by the Fuller grading equation. The shape coefficient *M* of crushed rocks ranges from 1.5 to 4.0, with an average value of 2.231.

### 2.2 Test design

The grading and shape distribution of rigid blocks follow the distribution shown in Fig 2(A) and 2(B) respectively. The size range of crushed rocks is 0.1 to 1.2 m. It is important to note that in order to balance simulation accuracy and computational efficiency, the shape of the actual crushed rock is simplified to reduce the number of invalid contacts between irregular rigid blocks [26, 27].

The 52304 fully-mechanized mining face of Daliuta Coal Mine in Shenmu County, Shaanxi Province, is taken as the research object, and the height of its caving zone is about 15 m. Therefore, in these numerical tests, the goaf was modeled as a unit of crushed rock measuring 15 m × 15 m × 15 m, which is approximately 20 times the average size [19]. The friction coefficients of the wall and rigid block, as shown in Table 1, were determined to be 0.50 and 0.55, respectively, based on the results of natural repose angle tests. The normal and shear stiffness of rigid blocks were set as 5.0e8 N/m, based on the suggestions given by Pierce and Cundall [28]. They demonstrated that stiffness only affects the running speed of the model, having a negligible influence on mechanical loading of fragmented rock. And the stiffness of wall should generally be slightly greater than the stiffness of rigid blocks to avoid the error of particle ‘going through the wall’. Thus, the normal and shear stiffness of wall were set as 1.0e9 N/m.

**Table 1:** Meso-mechanical parameters of wall and rigid block.

Wall			Rigid block			
Normal stiffnesses/ (N·m^−1^)	Shear stiffness/ (N·m^−1^)	Friction coefficient	Normal stiffnesses/ (N·m^−1^)	Shear stiffness/ (N·m^−1^)	Density/ (kg·m^−3^)	Friction coefficient
1 × 10^9^	1 × 10^9^	0.50	5 × 10^8^	5 × 10^8^	2300	0.55

### 2.3 Test procedures

**Fig 3:**
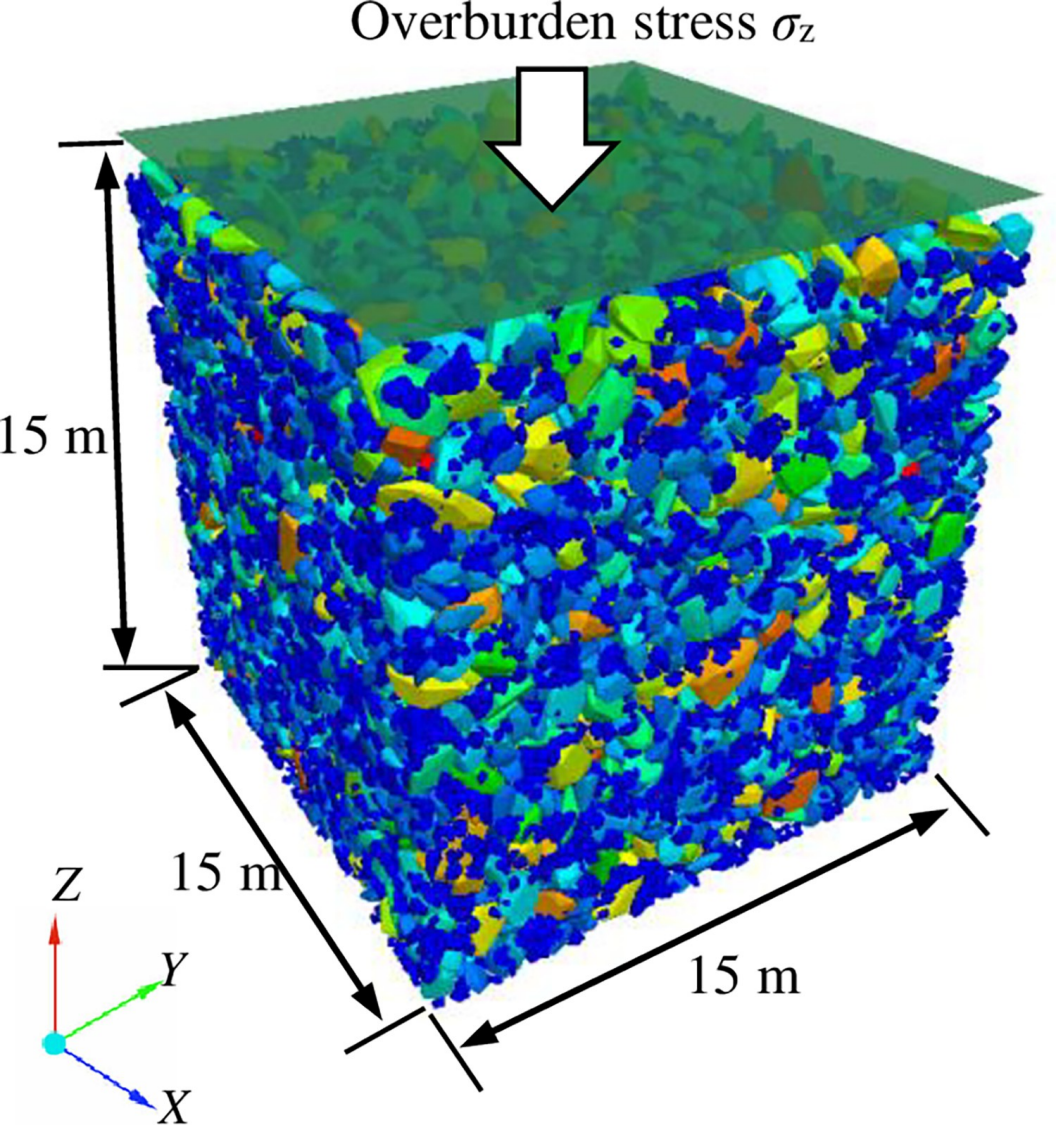
Initial numerical packing model of crushed rock.

Numerical compression tests of crushed rock with different overburden stresses *σ*_z_ were conducted as follows:

(1) Initial packing: Based on the linear contact model and after gravity settling [29], an initial packing model of crushed rock was created, and the walls and rigid blocks were assigned with the meso-mechanical parameters as presented in Table 1.

(2) Applying overburden stress: The side and bottom walls of the model were fixed, and the top wall moved quasi-statically along the negative direction of the *Z* axis at a speed of 0.002 m/s to compress the packing model of crushed rock. At the same time, the average stress on the top wall was monitored to stop compression when it reached 0, 2.5, 5, 7.5, 10, 12.5, 15, 17.5, 20 MPa (corresponding to the buried depth of about 800 m), respectively.

(3) Statistics of structural and mechanical parameters: Based on FISH language program, the average void ratio (storage coefficient), average coordination number and average contact force of packing system of crushed rock under different overburden stresses were counted, and the packing model was divided into layers every 1 m from bottom to top to calculate the storage coefficient, average coordination number and contact force of each layer. Fig 3 shows the initial numerical packing model of crushed rock.

## 3 Analysis of numerical test results

Based on the above numerical test results, spatio-temporal evolution laws of storage coefficient of underground reservoir and contact network of crushed rock under different overburden stresses *σ*_z_ were analyzed in terms of storage coefficient, void network structure and contact network.

### 3.1 Storage coefficient

**Fig 4:**
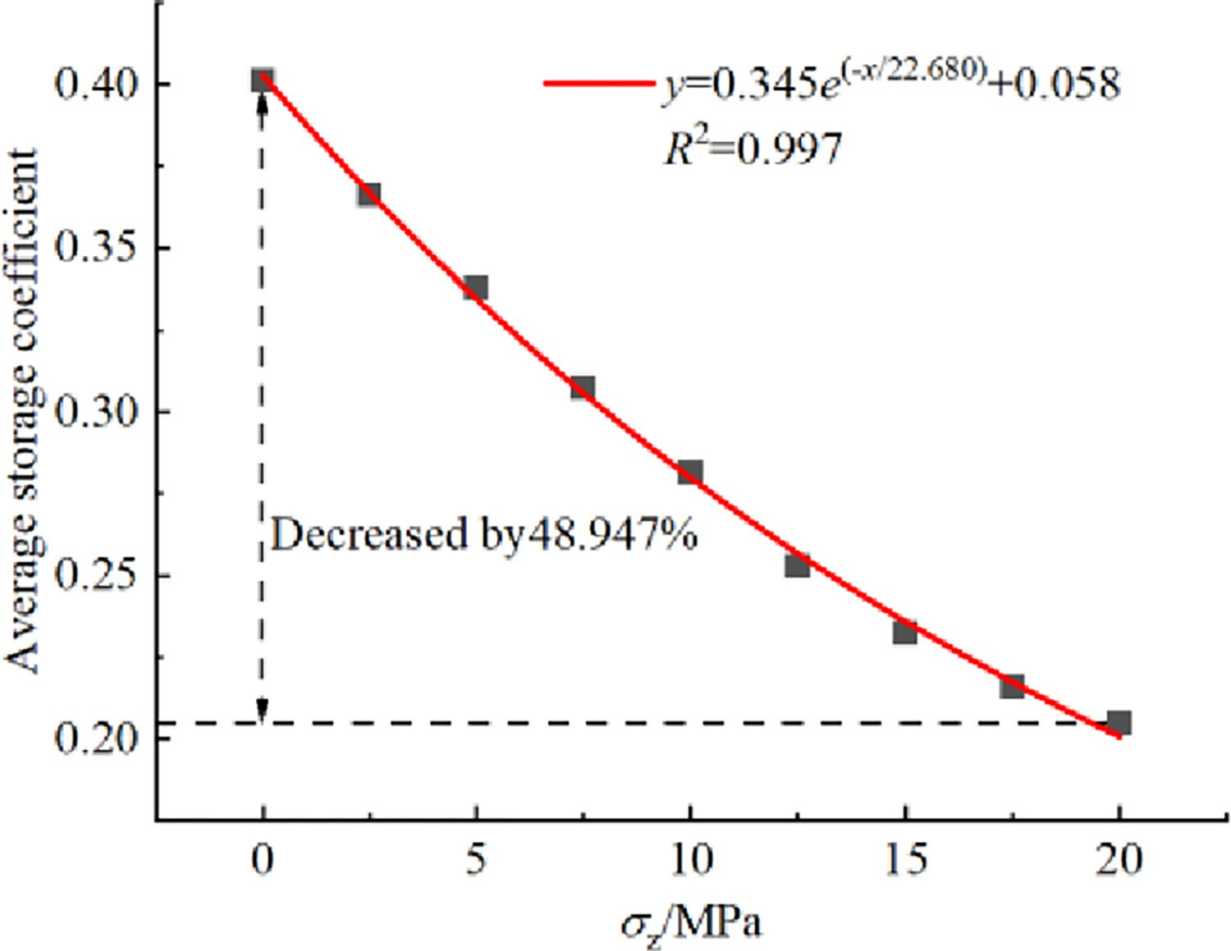
Relationship curve between average storage coefficient and overburden stress *σ*_z_.

The storage coefficient, which is the main parameter that characterizes the water storage capacity of underground reservoirs in coal mines, is equal to the void ratio of the packing model. The relationship between the average storage coefficient and overburden stress *σ*_z_ is shown in Fig 4. The analysis in Fig 4 reveals that the storage coefficient decreases exponentially as the overburden stress *σ*_z_ increases. When *σ*_z_ = 0, the storage coefficient is 0.403. However, when *σ*_z_ = 20 MPa, it decreases to only 0.205, indicating a decrease of 48.947%. The numerical compression tests mentioned above confirm that the general distribution range of the storage coefficient (0.2~0.4) aligns with the experimental value range of mine pumping and draining engineering (Fang et al. 2019), thus validating the reliability of the numerical compression model.

**Fig 5:**
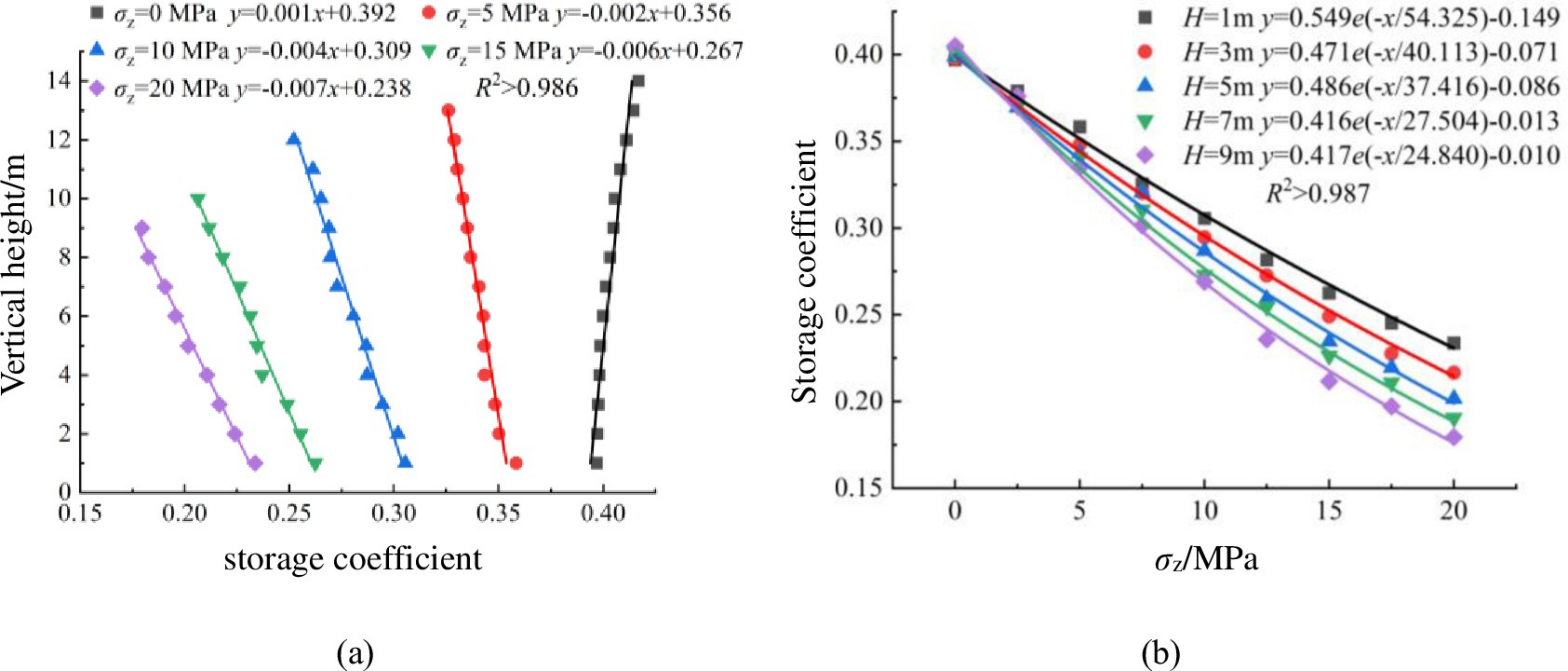
Distributions of storage coefficient at vertical height under different overburden stresses *σ*_z_: **a** variation of storage coefficient at vertical height, **b** variation of storage coefficient with overburden stress at different heights.

Fig 5 shows the distributions of storage coefficient at vertical height under different overburden stresses *σ*_z_. Since the height of packing model is 9.421 m at 20 MPa, only the storage coefficient within the height range of 9 m from the bottom of the model is considered in Fig 5(B). (1) As illustrated in Fig 5(A), when *σ*_z_ = 0, the storage coefficient of each layer of packing model increases linearly with the increase of vertical height *H*. When *σ*_z_ = 5, 10, 15 and 20 MPa, the storage coefficient decreases linearly with the increase of vertical height *H*, and the correlation coefficient *R*^2^ values are all above 0.986. And the larger the *σ*_z_ is, the greater the absolute value of the slope of the fitting straight line, indicating a more pronounced decreasing trend of the storage coefficient. The storage coefficient of the bottom layer and the top layer decreases by 8.962%, 17.470%, 21.408% and 23.170% respectively, indicating that the increase of overburden stress has great influence on the vertical distribution of storage coefficient. (2) As illustrated in Fig 5(B), the storage coefficient at different heights of the packing model decreases exponentially as the overburden stress increases, the correlation coefficient *R*^2^ values are all above 0.987. And as the vertical height *H* increases, the storage coefficient decreases, indicating that the overburden stress has a more significant impact on the upper storage coefficient in the packing system of crushed rock.

### 3.2 Void network structure

To examine the impact of various overburden stresses on the structure of water storage space, a void network model was employed to analyze the water storage space structure of crushed rock. The void network model is an equivalent model for reconstructing the complex void space structure, where the void is simplified as a sphere, the throat is simplified as a thin rod, and the void space is simplified as a series of sphere-rod connected network structures [30], so as to analyze the structural characteristics of water storage space of crushed rock.

**Fig 6:**
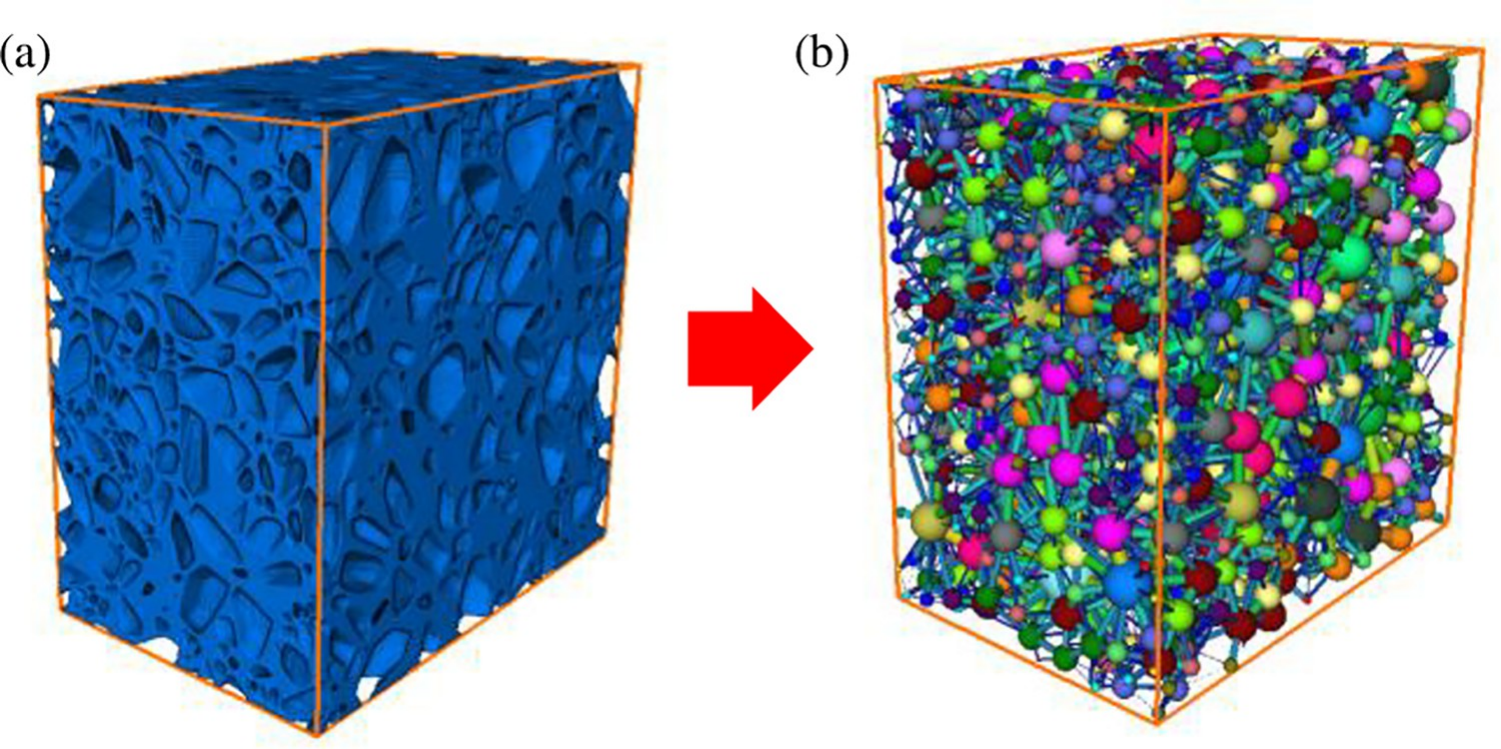
The void network model of water storage space of crushed rock: **a** water storage space of crushed rock, **b** void network model.

The Maximal Ball method [31] was employed to obtain the void network model of water storage space. As shown in Fig 6, voids and throats were divided by setting a sphere radius ratio of 0.7. If a maximum sphere radius was 0.7 times or more of the maximum sphere radius of its previous generation, it was identified as a void. Vice versa, if it was less than 0.7 times, it was considered as a throat [31]. And the minimum segmentation threshold was set to 0.02 m (about 1/5 of the smallest particle size). The main characteristic parameters of the void network model, such as void radius, void shape factor, and throat radius, were calculated and analyzed. The void radius indicates the size of the void and primarily influences the storage coefficient. The throat radius represents the size of the connecting channel between the voids and determines their connectivity. The void shape factor *G* indicates the degree of irregularity in the void space. A larger void shape factor indicates a more regular void shape. This parameter affects the manner and speed of fluid flow in the water storage space. Its calculation equation is as follows:

$$G=\frac{VL}{A_{s}}$$ (3)

Here, *V* is the void volume; *L* is the void length; *A*_s_ is the surface area of void.

**Fig 7:**
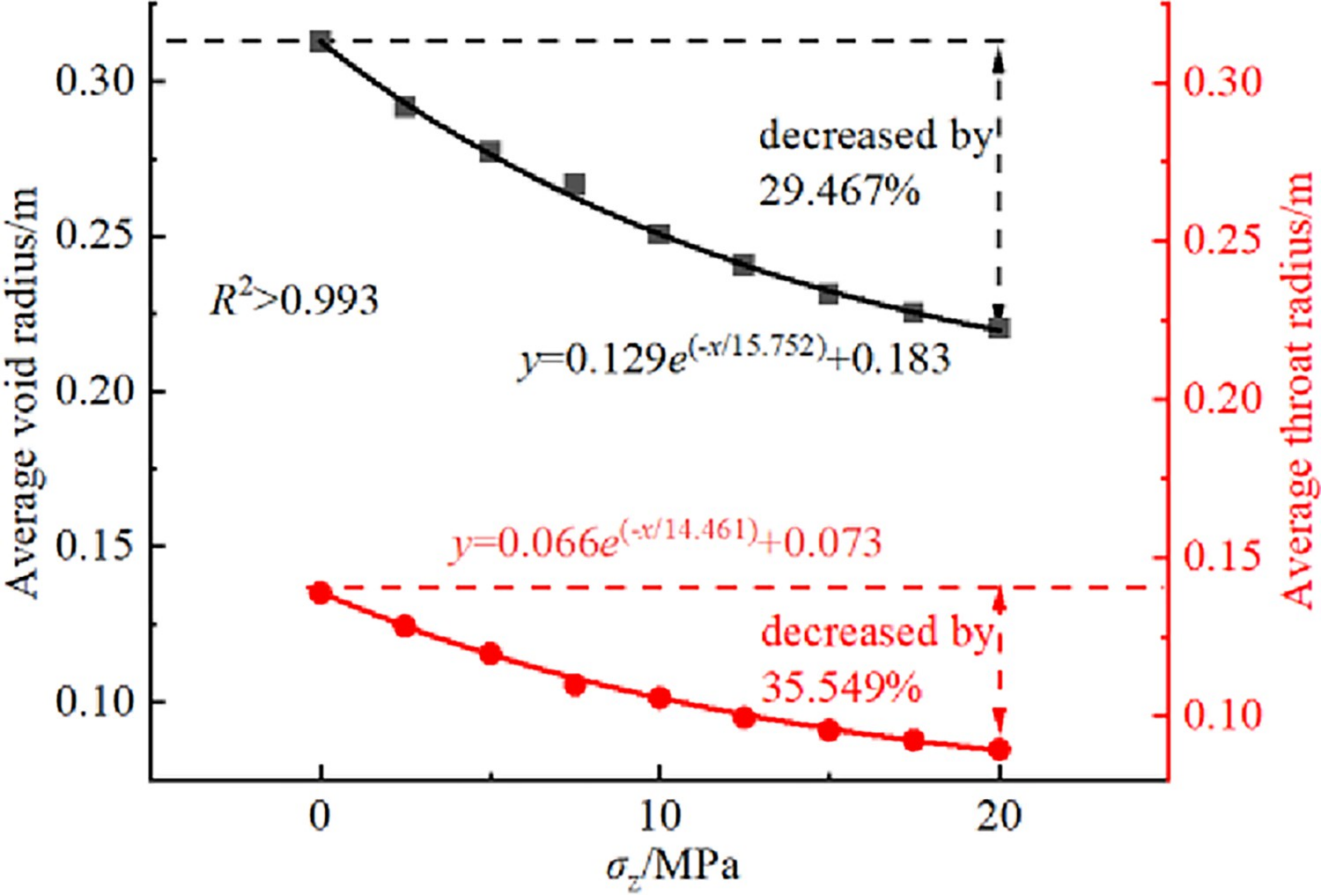
Variation curves of average void radius and average throat radius under different overburden stresses *σ*_z_.

The variation curves of average void radius and throat radius under different overburden stresses *σ*_z_ are shown in Fig 7. The analysis in Fig 7 reveals that the average void radius and average throat radius both decrease exponentially as the overburden stress increases. From *σ*_z_ = 0 to 20 MPa, the decreases are 29.467% and 35.549% respectively. This indicates that an increase in overburden stress results in a decrease in void radius and intervoid connectivity.

**Fig 8:**
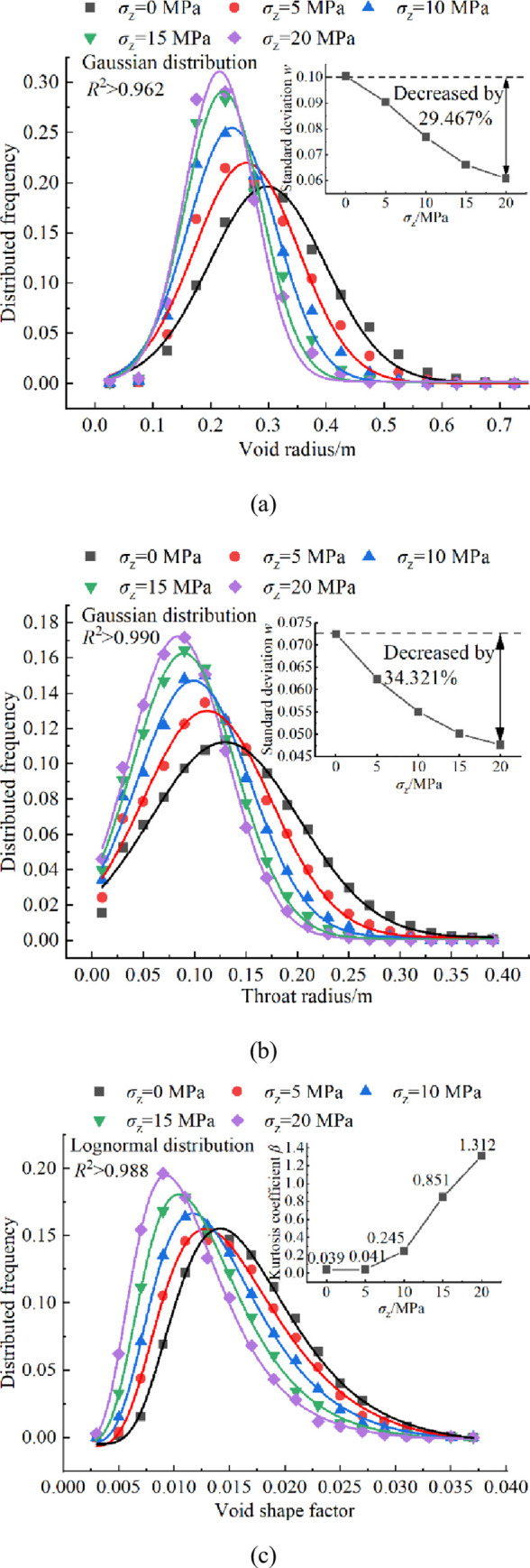
Distributions of characteristic parameters of void network model under different overburden stresses *σ*_z_: **a** void radius, **b** throat radius, **c** void shape factor.

Taking packing model with overburden stresses *σ*_z_ = 0, 5, 10, 15 and 20MPa as examples, the void network model was constructed and the distribution of its main characteristic parameters were calculated. The results are shown in Fig 8. The comparative analysis in Fig 8(A)–8(C) reveals that: (1) Under varying overburden stresses, the void radius and throat radius of the water storage space follow a Gaussian distribution, and the correlation coefficient *R*^2^ values are 0.962 and 0.990, respectively. With the increase of overburden stress, the peak range of the void radius and throat radius distribution curve narrows, and the peak value gradually decreases. Furthermore, the standard deviations of the fitting curve *w* decrease by 29.467% and 34.321% from *σ*_z_ = 0 to 20 MPa, respectively. This indicates that the anisotropy of void radius and throat radius distributions increases, and it implies that as the overburden stress increases, the packing system of crushed rock undergoes continuous compaction. Consequently, the internal large voids gradually transform into small voids, leading to a gradual increase in the proportion of small voids, the average void radius, and the radius of the connecting channel between voids. (2) The void shape factors all follow logarithmic normal distribution, and the correlation coefficient *R*^2^ values are all greater than 0.988. The increase of overburden stress leads to a narrowing of the peak range of the distribution curve, a gradual decrease in the peak value, and an increase in the kurtosis coefficient *β* from 0.039 to 1.312, as *σ*_z_ increases from 0 to 20 MPa. Noted that as the overburden stress increases from 0 MPa to 5 MPa, the peak value of the distribution frequency of void shape factor is not significantly different (red and black curves in Fig 8(C)), which is different from all other cases, such as void radius, throat radius. This indicates that compared to the void radius and throat radius, when the overburden stress is small (such as 0~5 MPa), the peak value of the void shape factor is not very sensitive to the changes in overburden stress, while when the overburden stress is relatively large (such as 5~20 MPa), the peak value of the void shape factor increases with the increase of overburden stress. Additionally, the anisotropy of the void shape factor distribution also increases, indicating that the void shape of the packing model becomes more irregular with the increase of overburden stress. The existing research indicates that small voids are generally more complex, whereas large voids tend to be regular [32, 33]. Furthermore, an increase in overburden stress results in a higher proportion of small voids, leading to irregular shapes of the overall voids. In conclusion, it can be inferred that overburden stress influences the storage coefficient by impacting the average void radius. Additionally, it affects the structure and connectivity of water storage spaces by influencing the distribution of void radius, throat radius, and void shape factor.

### 3.3 Contact network of crushed rock

Contact network is the physical basis of the external stress transmission path of particle system [34]. Analyzing the evolution law of contact networks is crucial for understanding the meso-mechanism of storage coefficient evolution. This study aims to analyze the meso-mechanical mechanism of spatial structure evolution in water storage spaces under varying overburden stresses. The analysis focuses on three aspects: the magnitude, quantity, and direction distribution of contact forces.

#### 3.3.1 Magnitude of contact force

**Fig 9:**
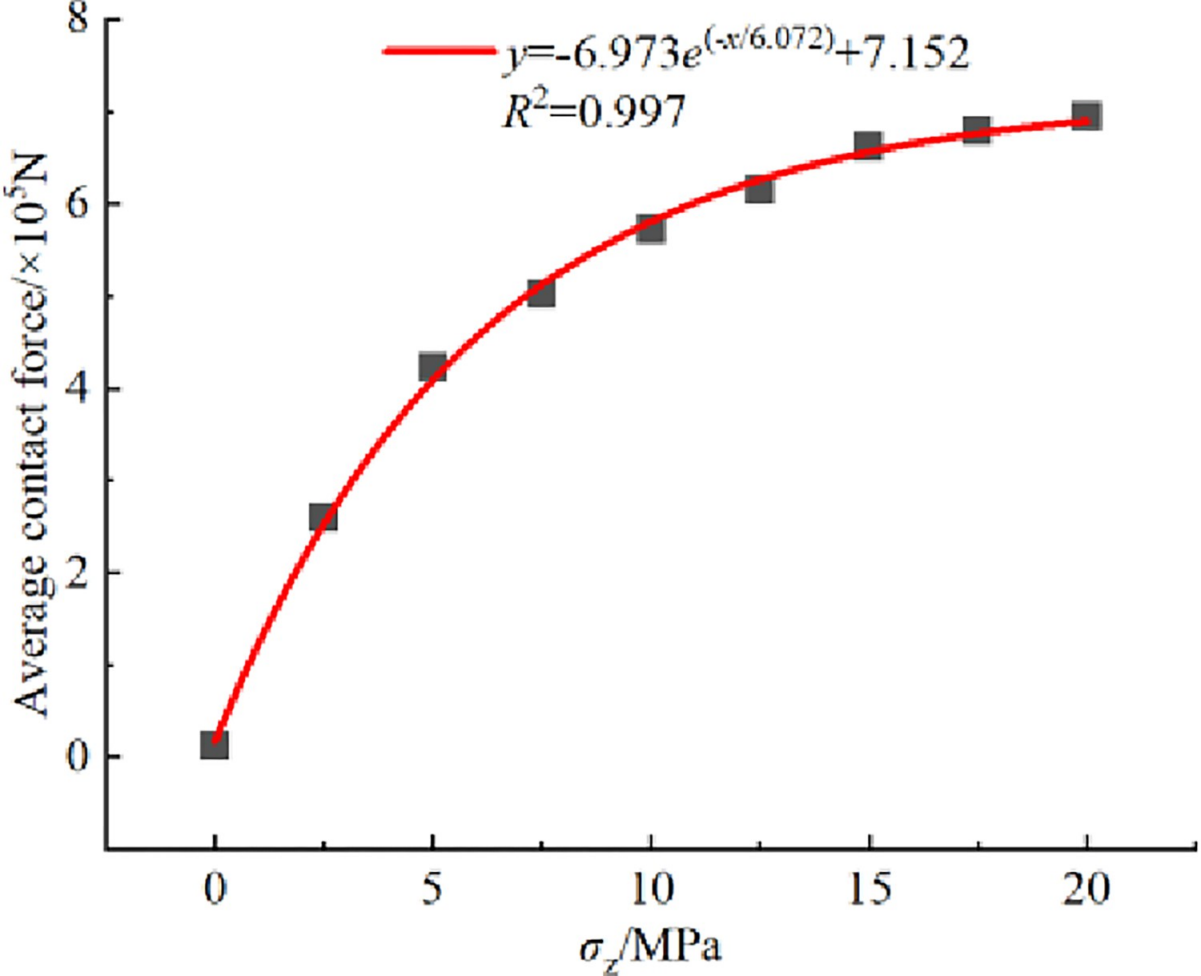
Relationship curve between average contact force of crushed rock and overburden stress *σ*_z_.

The relationship curve between average contact force of crushed rock and overburden stress *σ*_z_ is shown in Fig 9. The analysis in Fig 9 reveals that the average contact force of crushed rock increases exponentially with the increase of overburden stress *σ*_z_. For instance, when *σ*_z_ = 0, the average contact force is only 1.258×104 N. However, when *σ*_z_ = 20 MPa, the average contact force significantly increases to 6.950×105 N. This observation also provides an explanation for the exponential decrease in the storage coefficient shown in Fig 4 as the overburden stress *σ*_z_ increases.

**Fig 10:**
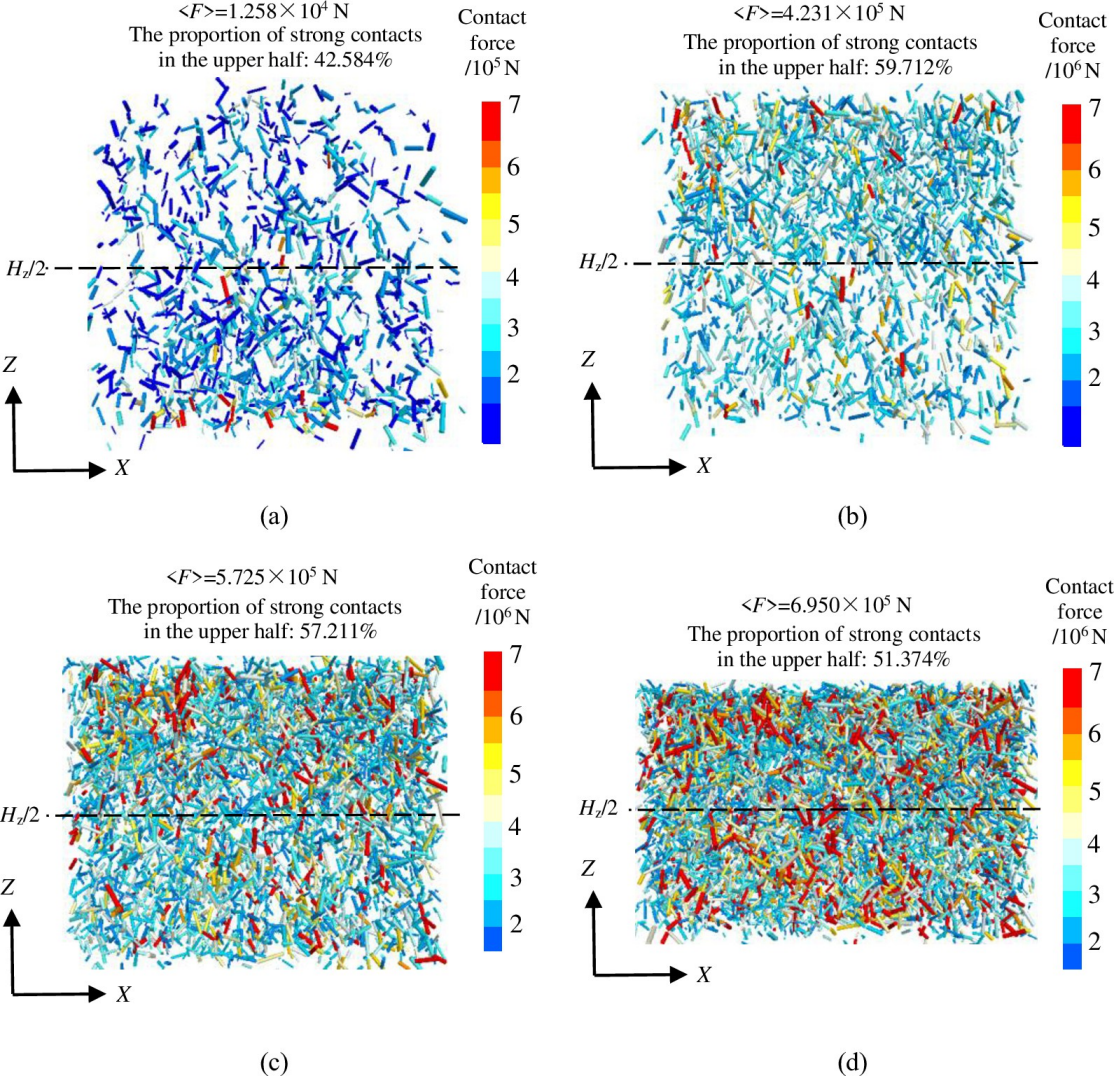
Contact distribution of the *XZ* section in the center of packing system of crushed rock: **a** *σ*_z_ = 0 MPa, **b** *σ*_z_ = 5 MPa, **c** *σ*_z_ = 10 MPa, **d** *σ*_z_ = 20 MPa.

In general, contacts that have a greater contact force than the average contact force of the packing system are considered strong contacts, while contacts with a lower contact force are considered weak contacts [35]. The three-dimensional contact distribution of the *XZ* section in the center of packing system of crushed rock is shown in Fig 10. (1) When *σ*_z_ = 0 (Fig 10(A)), the packing model exhibits a relatively small number of strong contacts, with only 42.584% of these strong contacts being present in the upper half. Consequently, the crushed rocks in the lower half of the packing model experience a greater amount of stress. (2) With the increase of overburden stress, the number of strong contacts increases and gradually expands to the bottom of the packing model (Fig 10(B)–10(D)). The proportion of strong contacts in the upper half is more than 50% and gradually decreases, which is 59.712%, 57.211% and 51.374%, respectively when *σ*_z_ = 5 MPa, 15 MPa and 20 MPa. The increase in vertical height leads to a larger proportion of strong contacts and average contact force in the lower half of the packing model, due to the gravity effect. This results in an increase in the water storage coefficient (Fig 5(A)). However, when considering the overburden stress, the stress on the crushed rocks is much greater than the stress generated by its own weight (less than 0.5 MPa) [20]. As a result, the number of strong contacts and average contact force in the upper half of the packing model are larger, leading to a decrease in the storage coefficient with increasing vertical height (Fig 5(A)).

**Fig 11:**
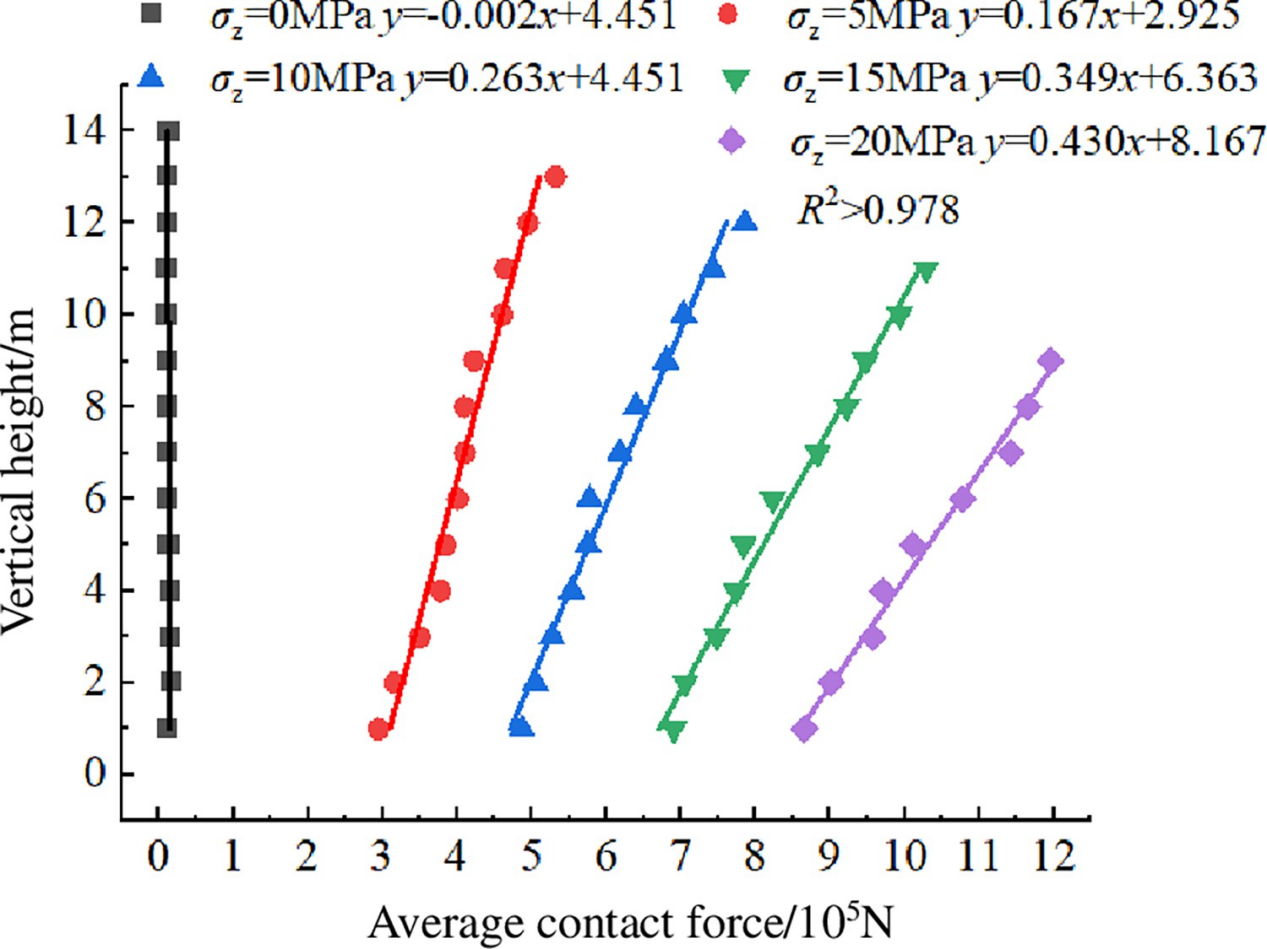
Average contact force variation curves of crushed rock under different overburden stresses *σ*_z_.

The variation curves of average contact force of crushed rock at vertical height under different overburden stress *σ*_z_ is shown in Fig 11. When *σ*_z_ = 0, there is no obvious difference in the average contact force of each layer in the packing system of crushed rock. When *σ*_z_ = 5 to 20 MPa, the average contact force of each layer increases linearly with the increase of vertical height. The slope of the fitting straight line becomes larger as the overburden stress increases. Compared with the lowest layer and the top layer in the packing system of crushed rock, the average contact force increases by 80.781%, 61.364%, 48.979%, and 38.086% respectively. This variation in the average contact force of crushed rock with vertical height is consistent with the linear variation trend of the storage coefficient shown in Fig 5(A).

#### 3.3.2 Coordination number

**Fig 12:**
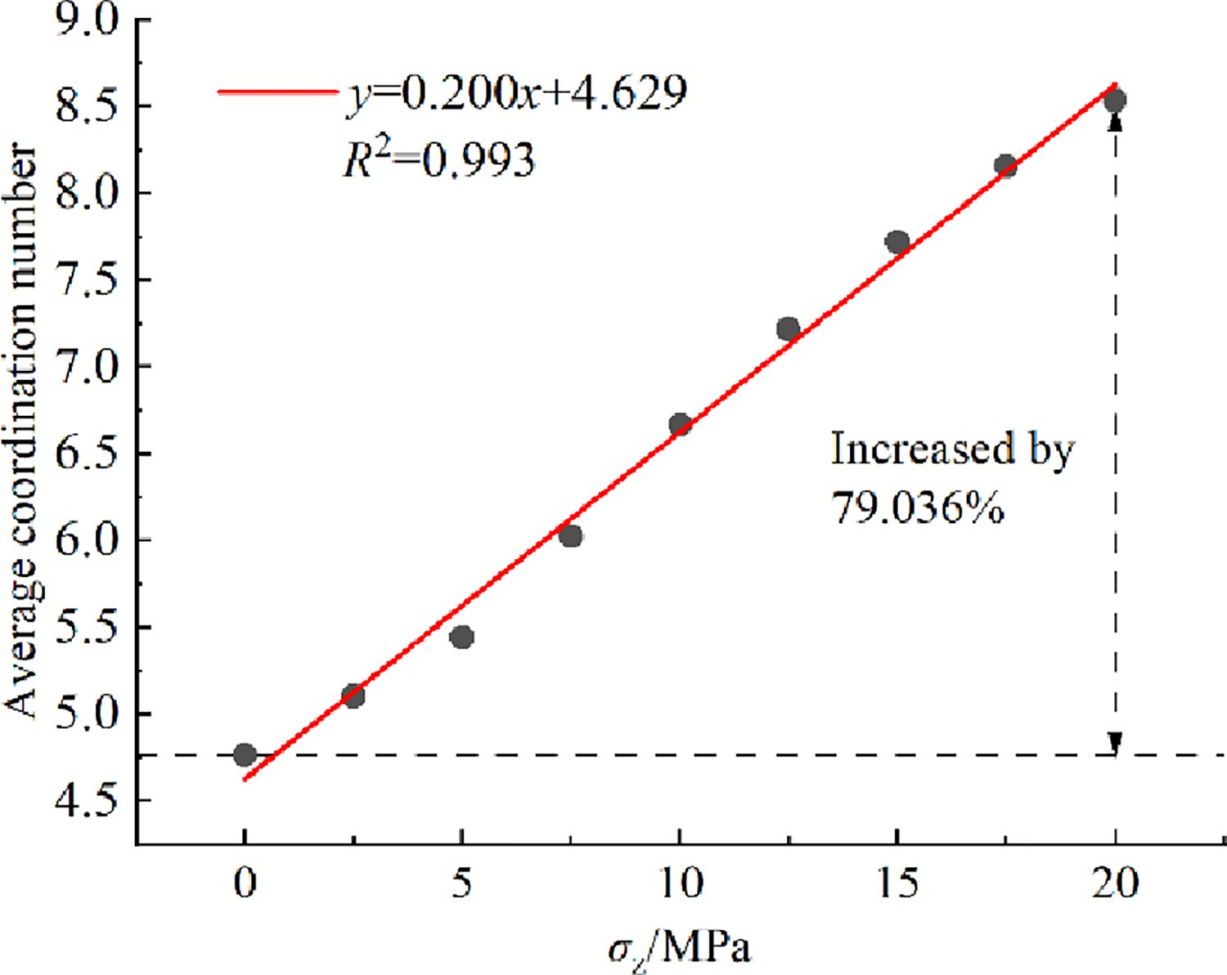
Relationship curve between average coordination number of crushed rock and overburden stress *σ*_z_.

Coordination number (number of contacts between particles) indicates the density of contact network of crushed rock. The relationship curve between average coordination number of crushed rock and overburden stress *σ*_z_ is shown in Fig 12. The analysis in Fig 12 reveals that the average coordination number increases linearly from *σ*_z_ = 0 to 20 MPa, with an increase of 79.036%.

**Fig 13:**
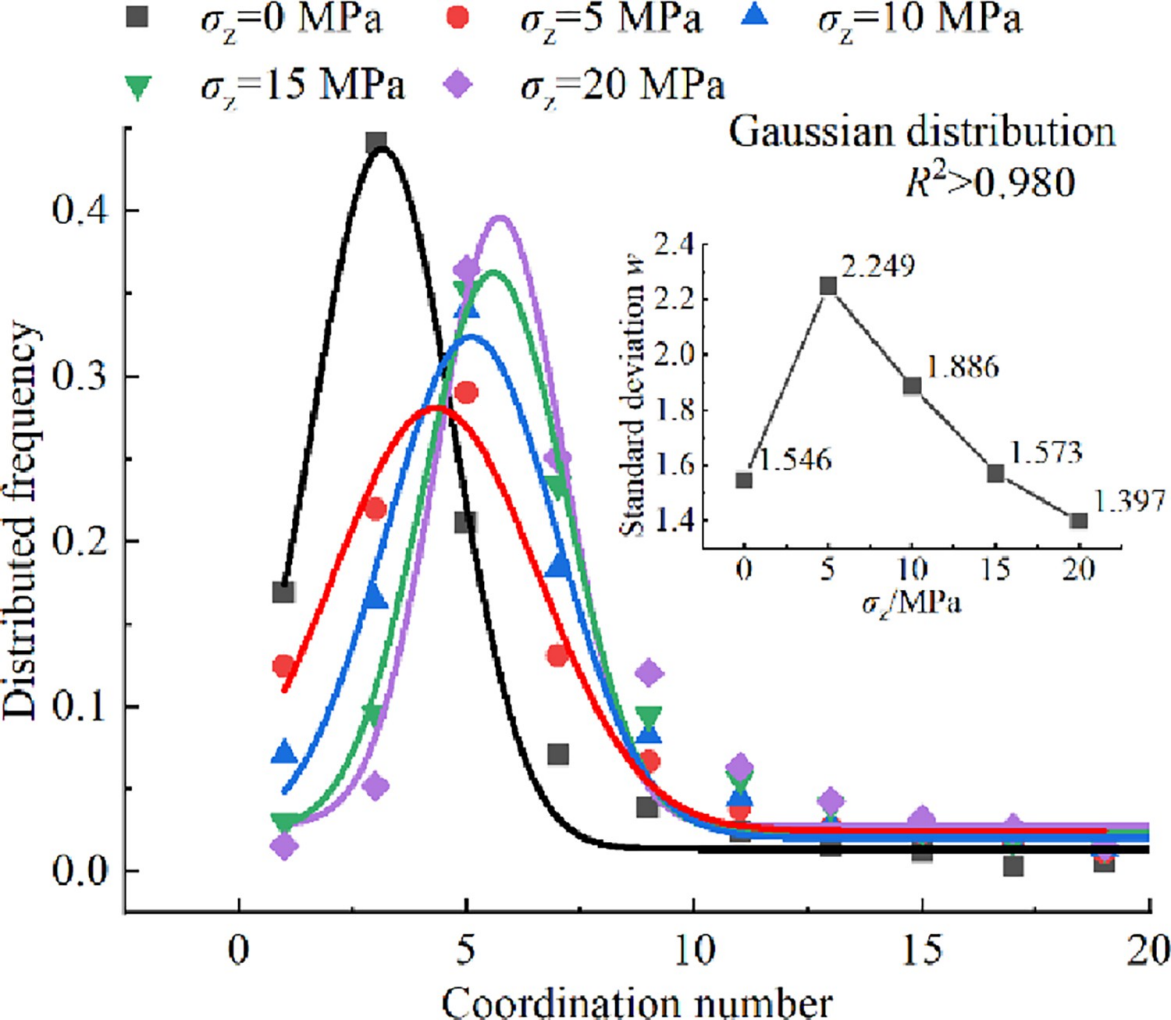
Distributed frequencies of coordination number of crushed rock under different overburden stresses *σ*_z_.

The probability density distribution of coordination number is a typical mechanical parameter to describe the heterogeneity and anisotropy of particle packing system structure. The distributed frequencies of coordination number of crushed rocks under the influence of different overburden stresses *σ*_z_ is shown in Fig 13. The distributions of coordination number of crushed rocks under the influence of different overburden stresses *σ*_z_ follow the Gaussian distribution, and the correlation coefficient *R*^2^ values are all greater than 0.980. When *σ*_z_ = 0, the peak coordination number of crushed rocks is approximately 3.0, accounting for 44.097%. The standard deviation *w* is 1.546, indicating that the packing model of crushed rock is relatively loose without overburden stress. As the overburden stress *σ*_z_ increases from 5 to 20 MPa, the peak value of the fitting curve gradually increases and the range of the peak value narrows. The standard deviation *w* decreases from 2.249 to 1.397, a decrease of 37.884%. This suggests that higher overburden stress leads to a narrower distribution range of coordination number of crushed rocks and an increased anisotropy of the packing system structure. Consequently, the void radius, throat radius, and peak value of void shape factor gradually decrease, and the range of peak values narrows (Fig 8).

#### 3.3.3 Direction of contact force

The contact normal direction of particles is defined as the unit vector of the contact direction between particles, and its spatial distribution can be quantitatively characterized by fabric tensor [36]:

$$\phi_{\mathrm{ij}}=\frac{1}{N_{c}}\sum_{1}^{N_{c}}n_{i}^{c}n_{j}^{c}$$ (4)

Here, *ϕ*_ij_ is the contact normal fabric tensor, $n_{i}^{c}$ is the unit vector along the contact normal direction, and *N*_*c*_ is the total contact number of the particle packing system. The second-order anisotropy tensor of the contact normal fabric tensor is unsymmetrical, and the degree of contact normal anisotropy $\alpha_{\mathrm{ij}}^{c}$ can be characterized by Eq (5):

$$\alpha_{\mathrm{ij}}^{c}=\frac{15}{2}\phi_{\mathrm{ij}}^{'}$$ (5)

Here, $\phi_{\mathrm{ij}}^{\prime }$ is the partial tensor of *ϕ*_ij_.

**Fig 14:**
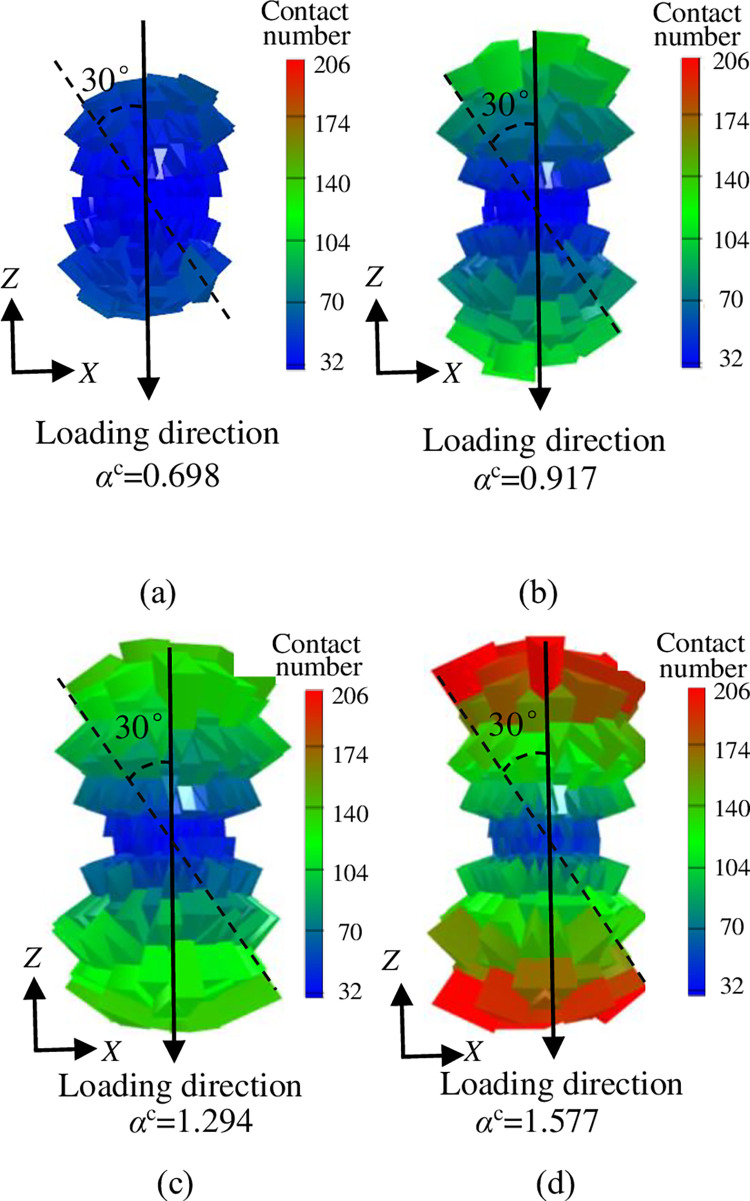
Three-dimensional rose diagrams of contact distribution of crushed rock: **a** *σ*_z_ = 0 MPa, **b** *σ*_z_ = 5 MPa, **c** *σ*_z_ = 10 MPa, **d** *σ*_z_ = 20 MPa.

The contact vector value of crushed rock was measured and the spatial position of the contact was calculated. The three-dimensional rose diagrams were created to illustrate the distribution of contact points under different overburden stresses (Fig 14). The analysis in Fig 14 reveals that the normal contact distribution has a shape similar to a peanut. Moreover, as the overburden stress increases, the majority of contact directions are concentrated within the ±30° range in the loading direction. The number of contacts and the contact normal anisotropy coefficient *α*^c^ also increase. Specifically, when *σ*_z_ = 0 MPa, *α*^c^ is only 0.698, but when *σ*_z_ = 20 MPa, *α*^c^ increases to 1.577, representing a 125.931% increase. This indicates that higher overburden stress results in a greater anisotropy in the distribution of contact force directions, which in turn affects the anisotropy of the packing system structure of crushed rock.

## 4 Conclusion

In this study, we conducted 3D numerical compression tests using the discrete element method and an irregular rigid block model. Our main objective was to analyze the relationship between the meso-structure characteristics of underground reservoir water storage space and the contact network of irregular crushed rock under varying overburden stresses. The main conclusions of this paper are as follows:

(1) The average storage coefficient and the storage coefficient at different vertical heights of the crushed rock packing system decrease exponentially as the overburden stress increases. In the range of this study, when *σ*_z_ = 0~20 MPa, the average storage coefficient decreases by 48.947%. Additionally, when *σ*_z_ = 5~20 MPa, the storage coefficient decreases linearly with the increase in vertical height.

(2) The average void radius and throat radius of water storage space decrease exponentially as the overburden stress *σ*_z_ increases. When *σ*_z_ = 0~20 MPa, the decreases are 29.467% and 35.549%, respectively. The increase in overburden stress leads to the transformation of large voids into smaller voids, causing a gradual decrease in void connectivity and a tendency towards irregular void shapes.

(3) The packing system of crushed rock experiences an increase in the number of strong contacts as the overburden stress increases. This phenomenon is observed to gradually expand from the top to the bottom of the model. Additionally, the average contact force of crushed rocks shows an exponential increase, while the coordination number increases linearly.

(4) As the overburden stress increases, the majority of contact directions are concentrated within the ±30° range in the loading direction. When the normal stress *σ*_z_ ranges from 0 to 20 MPa, the degree of contact normal anisotropy of crushed rocks increases by 125.931%. This increase results in an enhancement of the anisotropy of the packing system structure of crushed rock.

The influence of particle gradation, strength, breakage, model boundary conditions, and other factors on the storage coefficient, water storage spatial structure, mechanical characteristics, and the meso-control mechanism will be further investigated.
